# Prostate MRI vascular markers and erectile function after nerve‐sparing prostatectomy

**DOI:** 10.1002/bco2.70295

**Published:** 2026-10-08

**Authors:** Abdullah Al‐Mitwalli, Matthew Megson, Dalia Mohammed, Ahmed Al‐Nowfal, Conrad Von Stempel, Wai Gin Lee, David Ralph, Zafer Tandogdu

**Affiliations:** ^1^ Urology University College London Hospital London UK; ^2^ Division of Surgery and Interventional Sciences University College London London UK; ^3^ University College London Medical School London UK; ^4^ Radiology University College London Hospital London UK

**Keywords:** body mass index, erectile dysfunction, internal medicine, magnetic resonance imaging, medicine, prostate, prostate cancer, prostatectomy, retrospective cohort study, urology

## Abstract

**Objectives:**

To determine whether preoperative multiparametric magnetic resonance imaging (mpMRI)‐derived vascular markers are associated with baseline and postoperative erectile function (EF) and change in EF in men undergoing bilateral nerve‐sparing robot‐assisted radical prostatectomy (RARP).

**Patients and Methods:**

A secondary retrospective imaging analysis was undertaken using prospectively collected cohort data. Men undergoing bilateral nerve‐sparing RARP with a preoperative International Index of Erectile Function—erectile function domain score (IIEF‐6) and preoperative mpMRI incorporating dynamic contrast‐enhanced sequences were included. MRI‐detected accessory pudendal artery (APA), prostate enhancement ratio (PER) and corporal enhancement ratio (CER) were assessed by two expert uro‐radiologists. Associations with EF outcomes were examined using correlation analyses and univariable and multivariable linear regression.

**Results:**

Seventy‐one patients (mean age 60 years) met inclusion criteria; APA was identified in 31 (43.6%). APA presence was associated with higher baseline IIEF‐6 scores (mean 24.1 vs. 20.0, *p* = 0.043). Baseline IIEF‐6 demonstrated moderate negative correlation with PER (*ρ* = −0.30, *p* = 0.014) and moderate positive correlation with CER (*ρ* = 0.30, *p* = 0.018). On multivariable analysis, APA presence (*β* = 5.20, *p* = 0.019), PER (*β* = −0.049, *p* = 0.017) and CER (*β* = 0.040, *p* = 0.049) independently predicted baseline EF. Increasing age independently predicted lower baseline IIEF‐6 (*β* = −0.34, *p* = 0.017), while higher body mass index correlated with poorer 24‐month IIEF‐6 scores (*ρ* = −0.27, *p* = 0.046). Bilateral intrafascial/intra‐interfascial nerve‐sparing techniques were associated with superior 24‐month EF outcomes (mean 16.8 vs. 10.5, *p* = 0.028). No MRI‐derived marker predicted 24‐month EF or EF change.

**Conclusion:**

Preoperative mpMRI‐derived vascular markers, particularly APA identification, independently correlate with baseline EF in men undergoing BNS RARP, supporting a contributory vascular component in post‐prostatectomy ED. Prospective studies are warranted to determine whether intraoperative vascular preservation strategies, guided by preoperative MRI vascular mapping, influence functional recovery and can be integrated into surgical planning pathways.

## 1 INTRODUCTION

Robot‐assisted radical prostatectomy (RARP) has become the main surgical curative option for localized prostate cancer, the most common cancer in men worldwide.^1^, ^2^, ^3^ Nerve‐sparing radical prostatectomy aims to preserve the structures that provide parasympathetic nerves and vasculature in the neurovascular bundle on the posterolateral aspect of the prostate to improve erectile and urinary function after surgery.^4^ Despite advances in nerve‐sparing technique and contemporary surgical practice, including a high prevalence of bilateral nerve‐sparing RARP, postoperative erectile dysfunction (ED) remains a significant issue, with approximately 60% to 80% of men experiencing significant ED at 12 months.^5^, ^6^, ^7^ Erections are a haemodynamic process that occurs due to smooth muscle relaxation in the corpus cavernosum which requires intact nerves to initiate the relaxation and intact arterial vasculature to provide oxygenated blood to produce an erection.^8^, ^9^ Multiparametric magnetic resonance imaging (mpMRI) offers vasculature assessment of the pelvis in patients undergoing RARP.^10^ Therefore, we can leverage this to predict baseline and possibly postoperative erectile function (EF).

Anatomical variation in penile arterial supply is common, with an accessory pudendal artery (APA) contributing to penile arterial inflow in approximately one third of men and, in a minority, representing the sole source of arterial inflow.^11^, ^12^ The functional importance of APAs during radical prostatectomy remains uncertain. An earlier study suggested that APA preservation may facilitate postoperative EF recovery, whereas subsequent robotic series found no measurable adverse effect of APA transection. However, these studies relied on intraoperative identification without preoperative vascular mapping or postoperative confirmation of vessel integrity, limiting definitive conclusions regarding the functional benefit of APA preservation.^13^, ^14^, ^15^

mpMRI has become an integral component of the prostate cancer diagnostic pathway. Among its many advantages, mpMRI can predict post‐prostatectomy urinary incontinence outcomes through measurement of membranous urethral length (MUL). This imaging parameter not only serves as a prognostic tool for postoperative continence but is also a standard of care in preoperative patient counselling, helping to set realistic expectations for surgical outcomes.^16^

There is growing interest in identifying mpMRI‐derived markers to predict EF recovery following prostatectomy. In our recently published systematic review summarizing the literature on imaging predictors in patients undergoing robot‐assisted or laparoscopic radical prostatectomy, we found significant heterogeneity among studies and a generally high risk of bias.^10^ Nevertheless, we identified three key mpMRI‐based markers that may have potential prognostic value for EF recovery: (1) prostatic enhancement, (2) corporal enhancement and (3) MRI‐detected APAs. These markers can be extracted from the dynamic contrast enhancement (DCE) sequence of the mpMRI. These findings highlight the emerging role of mpMRI in preoperative EF prognostication, though further validation is needed.

In this study, we evaluated the ability of DCE MRI‐derived markers to predict baseline and postoperative EF in men with prostate cancer undergoing bilateral nerve‐sparing RARP, with particular focus on their potential role in informing preoperative counselling and surgical planning.

## 2 METHODOLOGY

### 2.1 Study population

We reviewed all consecutive patients undergoing RARP in 2021 at University College London Hospitals with prospectively collected data through an exploratory non‐intervention prospective study assessing outcomes after RARP.^17^ The original study was approved as an audit by University College London Urology (audit number: 2202009) and received ethical approval from Anglia Ruskin University, Faculty of Health, Medicine & Social Care, Faculty Research Ethics Panel (ref ETH2122‐0590).

We then carried out a secondary imaging review for these patients with our select inclusion criteria encompassing patients who underwent bilateral nerve‐sparing surgery, had completed preoperative and 24‐month postoperative International Index of Erectile Function—erectile function domain (IIEF‐EFD also known as IIEF‐6) questionnaires and had undergone preoperative mpMRI with DCE. Exclusion criteria included poor‐quality MRI, biparametric MRI (non‐contrast studies) and any prior treatment for prostate cancer, including focal treatment, radiotherapy or androgen deprivation therapy. IIEF‐6 scores were recorded and analysed irrespective of the concurrent use of erectogenic therapies and therefore represent overall EF achieved with or without pharmacological assistance.

### 2.2 Radiological assessment and MRI markers

All MRI studies were assessed retrospectively after RARP by two expert uro‐radiologists (C.V.S. and A.A.‐N.) based at a high‐volume referral centre, with extensive experience in performing prostate artery embolization and embolization for high‐flow priapism. Together, they manage an annual caseload of 70 prostate artery embolization procedures per year. This study utilized the pre‐existing preoperative mpMRI, and no additional imaging was acquired specifically for this study. No postoperative imaging was performed.

MRI scans were included from all quality‐controlled MRI scanners within the prostate cancer network (Pi‐QAL).^18^ The magnet strength (1.5 or 3.0 T) and imaging quality were assessed according to predefined bespoke criteria. Minimum acceptable quality included full field‐of‐view coverage from the base of the prostate to the mid‐shaft of the corpora cavernosa and adequate contrast enhancement sequences including a minimum data set of fat saturated unenhanced T1w, arterial phase FST1w (within 30 s of contrast administration) with internal iliac and terminal branch enhancement without prostatic parenchymal enhancement and serial DCE sequences up to 15 min.

### 2.3 Vascular anatomy assessment

MRI studies were independently reviewed for the presence of APA by the two expert radiologists, who were blinded to surgical details, EF scores and each other's assessments. At final analysis, all discrepancies between readers were discussed and resolved by consensus.

APAs were documented if visualized. Their presence, origin and contribution to penile vascular supply were described.

### 2.4 Vascular index measurements on MRI: Enhancement ratios of the prostate and corpora

Both prostatic and corporal enhancement ratios (PER and CER) were calculated as the change in signal intensity from baseline to peak enhancement divided by the baseline signal intensity. All DCE sequences were acquired as part of routine diagnostic preoperative prostate MRI, with the penis in the flaccid state and without pharmacological erectile stimulation.

#### 2.4.1 PER^19^

A region of interest (ROI) was placed in the transition zone of the prostate, deliberately away from any visible pathology. Signal intensity was recorded at four key time points: baseline (at baseline and before contrast), initial vascular enhancement (marked by opacification of the external and internal iliac arteries), first visible prostatic enhancement and peak enhancement.

#### 2.4.2 CER^20^

ROIs were placed on the right corpus cavernosum to assess perfusion dynamics. Signal intensity was measured at the same four time points outlined above.

PER and CER were therefore treated as semi‐quantitative measures of resting tissue contrast enhancement rather than direct measurements of blood flow. In particular, CER was not intended to quantify stimulated cavernosal arterial inflow or corporo‐venocclusive function.

### 2.5 Statistical analysis

All statistical analyses were conducted using R software (R Foundation for Statistical Computing, Vienna, Austria). Descriptive statistics were used to summarize baseline characteristics.

Interobserver variability in the detection of APA was assessed using Cohen's kappa coefficient, with values interpreted according to Landis and Koch's criteria.

Comparisons of baseline and 24‐month postoperative IIEF‐6 scores between binary groups (e.g., presence or absence of APA, diabetes, hypertension and smoking status) were assessed using Welch's two‐sample *t*‐tests. For categorical variables with more than two levels (e.g., nerve‐sparing type), one‐way ANOVA or Kruskal–Wallis tests were applied, depending on data normality assessed using the Shapiro–Wilk test. Where appropriate, categories were collapsed (e.g., nerve‐sparing groupings) for binary comparisons.

Correlation analyses between continuous variables and IIEF‐6 scores at baseline and 24 months were conducted using Spearman's rank correlation coefficient (*ρ*), with 95% confidence intervals (CIs) computed via Fisher's *z*‐transformation.

Univariable linear regression models were first constructed for each of three outcomes including baseline and 24 monthly IIEF‐6 and ΔIIEF‐6 (difference baseline minus 2 yearly IIEF‐6).

Independent variables included clinical factors such as age, body mass index (BMI), hypertension, diabetes, dyslipidaemia and smoking, while imaging‐derived markers (PER, CER and APA presence). For each regression, beta coefficients (*β*), 95% CIs and *p* values were reported.

Following univariable analyses, two separate multivariable linear regression models were models; one included only clinical variables (age, BMI, hypertension, diabetes, hypercholesterolemia, smoking and nerve‐sparing group). The second model incorporated both clinical variables and imaging markers (PER, CER and APA presence).

A *p* value < 0.05 was considered statistically significant.

## 3 RESULTS

Of the 151 eligible patients screened, 80 were excluded due to either having biparametric MRI (24) or poor‐quality mpMRI (56). A total of 71 patients were included in this study with mean age of 60 years (7.6). The mean preoperative and 24‐month IIEF‐6 scores were 22 (9) and 15.5 (10), respectively. Table 1 provides further demographic and clinical details. Twenty‐four‐month IIEF‐6 data were available for 56 of the 71 included patients. Of these 56 patients, 19 (33.9%) reported that they had not attempted sexual intercourse during the 4 weeks preceding completion of the IIEF‐6 questionnaire.

**TABLE 1:** Demographics, medical history, baseline and follow‐up IIEF‐6 scores and intraoperative nerve status.

Total number included in study	71
Ethnicity—White	37/71 (52%)
Ethnicity—Black	24/71 (34%)
Ethnicity—Asian	6/71 (8.5%)
Ethnicity—other/mixed	4/71 (5.6%)
Age	60 (7.6)
Body mass index (kg/m^2^)	27.8 (3.5)
Diabetic	10/71 (14%)
Smokers	30/71 (42%)
Hypertension	30/71 (42%)
Dyslipidaemia	24/71 (34%)
Baseline mean IIEF‐6 score (SD) *N* = 71	21.8 (9)
24 months mean IIEF‐6 score (SD) *N* = 56	15.5 (10)
Nerve sparing—intra/intra	37/71 (52%)
Nerve sparing—intra/inter	18/71 (25%)
Nerve sparing—inter/inter	16/71 (23%)

Of the 71 patients, 31 (43.6%) had APA detected with right, left and bilateral presence in 16 (23%), 13 (18%) and 2 (3%) patients, respectively. Of the 33 APAs identified, 15 were branches of the obturator artery (45%), 12 (36%) branches of the prostatic artery and 6 (18%) branches of the anterior division of the internal iliac as highlighted in Figure 1. The mean size of the APA was 1.9 mm (0.46). The percent agreement between readers was 90.1%, corresponding to a Cohen's kappa of 0.80, indicating substantial agreement according to the Landis and Koch criteria.

**FIGURE 1:**
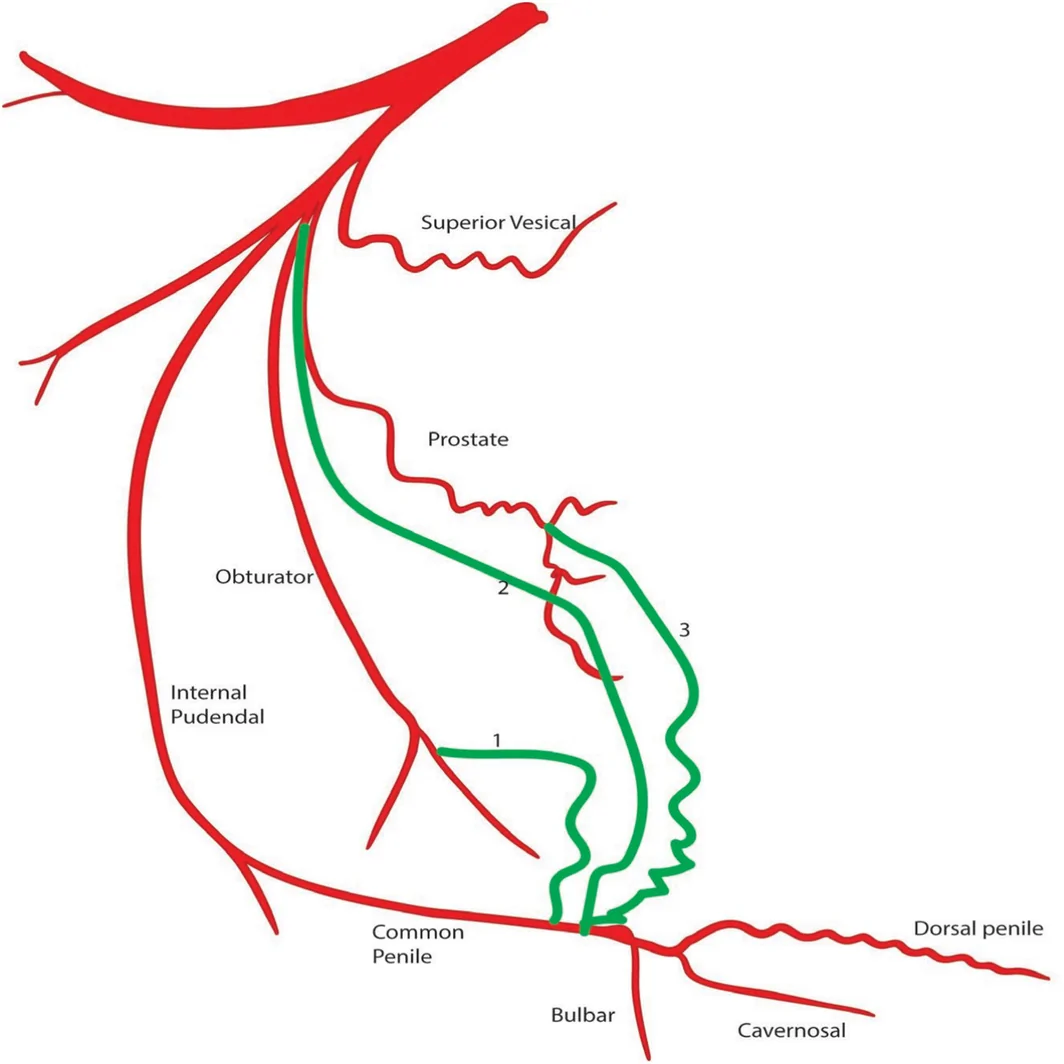
A schematic illustration of the pelvic vasculature. (1) corresponds to an APA arising from the obturator artery, which is a direct branch of the anterior division of the internal iliac artery; (2) corresponds to an APA branching directly from the anterior division of the internal iliac artery; and (3) corresponds to an APA arising from the prostatic artery.

Patients with an MRI‐detected APA demonstrated significantly higher baseline IIEF‐6 compared with those without an MRI‐detected APA (mean baseline IIEF‐6: 24.1 [7.5] vs. 20.0 [9.5], *p* = 0.043). However, there was no statistically significant difference in at 24 months postoperatively between the groups. Spearman's rank correlation demonstrated a moderate negative association between preoperative IIEF‐6 scores and PER that reached statistical significance (*ρ* = −0.3, *p* = 0.014) and a moderate positive association with CER (*ρ* = 0.3, *p* = 0.018). At 24 months postoperatively, there were no significant correlations between IIEF‐6 scores and either PER or CER (PER: *ρ* = −0.08, *p* = 0.56; CER: *ρ* = 0.05, *p* = 0.71). Table 2 summarizes the univariable analysis of clinical and imaging factors associated with EF outcomes.

**TABLE 2:** Univariate analysis table.

		Baseline IIEF (mean/Spearman's *ρ*)	*p* value	24‐month IIEF (mean/Spearman's *ρ*)	*p* value	IIEF‐6 delta (baseline scores–24‐month scores)	*p* value
APA	+	24.13 (SD 7.5)	**0.043**	15.6 (SD 10)	0.87	−8.8 (SD 12.1)	0.17
	−	19.98 (SD 9.5)		15.2 (SD 9)		−4.5 (SD 12)	
Hypertension	+	20.97 (SD 9.1)	0.51	14.48 (SD 9.7)	0.53	−6.5 (13)	0.95
	−	22.4 (SD 8.8)		16.12 (SD 9.5)		−6.3 (SD 10)	
Diabetes	+	18.8 (SD 10)	0.32	14.5 (SD 7.1)	0.71	−6.5 (SD 12)	0.82
	−	22.3 (SD 8.6)		15.6 (SD 9.9)		−5.6 (10.6)	
Dyslipidaemia	+	20.59 (SD 9.5)	0.44	13.85 (SD 8.4)	0.33	−8.4 (SD 9.7)	0.31
	−	22.4 (SD 8.6)		16.3 (SD 10.1)		−5.3 (SD 13)	
Smoking	+	21.17 (SD 8.9)	0.62	14.27 (SD 9.5)	0.4	−6.3 (SD 12.3)	0.96
	−	22.24 (SD 8.9)		16.47 (SD 9.6)		−6.5 (SD 11.6)	
PER		−0.295 (95% CI: −0.49, −0.06)	**0.014**	−0.083 (95% CI: −0.345, 0.192)	0.56	0.191 (95% CI: −0.084, 0.438)	0.17
CER		0.296 (95% CI: 0.05, 0.5)	**0.018**	0.053 (95% CI: −0.23, 0.33)	0.71	0.104 (95% CI: −0.374, 0.183)	0.48
Age		−0.297 (95% CI: −0.496, −0.068)	**0.012**	0.073 (95% CI: −0.195, 0.328)	0.6	0.222 (95% CI: −0.043, 0.459)	0.1
Body mass index		−0.097 (95% CI: −0.32, 0.139)	0.42	−0.27 (95% CI: −0.5, −0.006)	**0.046**	−0.21 (95% CI: −0.448, 0.056)	0.12
Nerve spare	Inter/inter			10.5 (SD 7.8)	**0.028**	−9.8 (SD 14)	0.35
	Intra/intra or intra/inter			16.8 (9.7)		−5.5 (11.1)	

Abbreviations: APA, accessory pudendal artery; CER, corporal enhancement ratio; PER, prostate enhancement ratio.

*Note*: Values are group means for categorical variables and Spearman's correlation coefficients (*ρ*) for continuous variables (PER and CER). *p* values for categorical variables were calculated using Welch's two‐sample *t*‐test. *p* values for continuous variables were derived from Spearman's rank correlation. Statistically significant *p* values are shown in bold.

Among the imaging variables, age demonstrated a significant association with baseline IIEF‐6 scores (*ρ* = −0.30, 95% CI: −0.50 to −0.07, *p* = 0.012). However, age was not significantly associated with 24‐month IIEF‐6 scores (*ρ* = 0.07, 95% CI: −0.20 to 0.33, *p* = 0.60), nor with EF change (ΔEF6) over time (*ρ* = 0.22, 95% CI: −0.04 to 0.46, *p* = 0.10). BMI did not correlate with baseline EF (*ρ* = −0.10, 95% CI: −0.32 to 0.14, *p* = 0.42) but was negatively associated with 24‐month IIEF‐6 scores (*ρ* = −0.27, 95% CI: −0.50 to −0.01, *p* = 0.046).

At 24 months, patients with bilateral intrafascial or intra/inter nerve‐sparing had higher mean IIEF‐6 scores 16.8 (9.7) compared with those with bilateral interfascial approaches with mean 10.5 (7.8); *p* = 0.028. Similarly, ΔEF6 was greater among patients with inter/inter nerve‐sparing compared with those with intra/inter or intra/intra techniques without reaching statistical significance. Other clinical factors, including hypertension, diabetes mellitus, hypercholesterolaemia and smoking status, did not demonstrate statistically significant associations with either baseline or postoperative IIEF‐6 scores.

On univariable linear regression, age was independently associated with lower baseline IIEF‐6 scores (*β* = −0.3, 95% CI: −0.57 to −0.03, *p* = 0.026), while MRI‐detected APA was significantly associated with higher baseline IIEF‐6 (*β* = 4.15, 95% CI: 0.006 to 8.3, *p* = 0.049). PER demonstrated a weak inverse association with baseline IIEF‐6 (*β* = −0.04, 95% CI: −0.08 to −0.005, *p* = 0.03). No other clinical or imaging markers were independently associated with 24‐month IIEF‐6 or ΔIIEF‐6 on univariable modelling as demonstrated in Table 3.

**TABLE 3:** Univariate linear regression model.

Predictor	Regression coefficient (*β*) baseline IIEF‐6 [95% CI]	*p* value	Regression coefficient (*β*) 24‐month IIEF [95% CI]	*p* value	Regression coefficient (*β*) IIEF‐6 delta [95% CI]	*p* value
Age	−0.3 [−0.57, −0.03]	**0.026**	0.10 [0.27, 0.47]	0.60	0.3 [−1.6, 0.76]	0.20
Hypertension	−1.4 [−5.7, 2.8]	0.51	−1.6 [−6.9, 3.9]	0.53	−0.23 [−6.7, 6.2]	0.94
Diabetes	−3.5 [−9.5, 2.5]	0.25	−1.1 [−8.5, 6.3]	0.77	0.92 [−8.2, 10]	0.84
Dyslipidaemia	−1.8 [−6.3, 2.6]	0.42	−2.5 [−7.8, 2.9]	0.36	−3.1 [−9.7, 3.5]	0.35
Smoking	−1.1 [−5.5, 3.2]	0.62	−2.2 [−7.3, 2.9]	0.40	0.16 [−6.3, 6.8]	0.96
Body mass index	−0.26 [−0.87, 0.35]	0.4	0.29 [−1, 0.46]	0.44	−0.18 [−1.2, 0.8]	0.72
Nerve group			**6.3 [0.24, 12.3]**	**0.042**	4.3 [−3.4, 12]	0.27
APA	+4.15 [0.006, 8.3]	**0.049**	−0.42 [−5.6, 4.8]	0.87	−3.1 [−9.5, 3.3]	0.34
PER	−0.04 [−0.08, −0.005]	**0.03**	−0.02 [−0.06, 0.03]	0.39	0.02 [−0.03, 0.08]	0.37
CER	0.029 [−0.01, 0.07]	0.16	0.03 [0.02, 0.74]	0.28	0.01 [−0.05, 0.07]	0.73

*Note:* Statistically significant *p* values are shown in bold.

Abbreviations: APA, accessory pudendal artery; CER, corporal enhancement ratio; PER, prostate enhancement ratio.

Multivariable regression was subsequently performed to determine whether the inclusion of imaging markers improved the explanatory power of clinical models. In the first model (clinical predictors only), increasing age remained a significant independent predictor of baseline IIEF‐6 (*β* = −0.31, 95% CI: −0.60 to −0.03, *p* = 0.029). None of the clinical predictors demonstrated significant associations with 24‐month IIEF‐6 or EF change. The summary of this is highlighted in Table 4.

**TABLE 4:** Multivariable linear regression results—Model A.

Predictor	Regression coefficient (*β*) baseline IIEF‐6 [95% CI]	*p* value	Regression coefficient (*β*) 24‐month IIEF [95% CI]	*p* value	Regression coefficient (*β*) IIEF‐6 delta [95% CI]	*p* value
Age	**−0.31 [−0.60, −0.03]**	**0.029**	0.19 [−0.22, 0.60]	0.358	0.40 [−0.12, 0.91]	0.128
Hypertension	−0.31 [−5.01, 4.40]	0.897	−1.52 [−7.48, 4.44]	0.610	0.69 [−6.72, 8.09]	0.853
Diabetes	−2.84 [−9.44, 3.76]	0.393	0.41 [−8.53, 9.36]	0.927	3.56 [−7.69, 14.8]	0.527
Dyslipidaemia	−0.07 [−5.23, 5.09]	0.979	−1.48 [−8.45, 5.49]	0.671	−5.01 [−13.9, 3.84]	0.261
Smoking	−1.41 [−5.68, 2.87]	0.514	−0.67 [−6.19, 4.85]	0.808	1.69 [−5.12, 8.50]	0.620
Body mass index	−0.28 [−0.91, 0.35]	0.383	0.04 [−0.82, 0.90]	0.926	0.21 [−0.89, 1.30]	0.706
Nerve group			6.50 [−0.53, 13.5]	0.069	5.29 [−3.33, 13.9]	0.223

*Note*: Summary of multivariable linear regression models predicting baseline erectile function, 2‐year erectile function and change in scores. Model A includes only clinical predictors. Statistically significant *p* values are shown in bold.

In the second model, which included both clinical and imaging variables, the adjusted *R* ^2^ for predicting baseline IIEF‐6 improved markedly from 0.019 in Model A to 0.188, with a substantial reduction in AIC (518.8 vs. 462.8). MRI‐detected APA (*β* = 5.20, 95% CI: 0.89 to 9.51, *p* = 0.019), PER (*β* = −0.049, 95% CI: −0.089 to −0.009, *p* = 0.017) and CER (*β* = 0.040, 95% CI: 0.0001 to 0.079, *p* = 0.049) all emerged as independent predictors of baseline EF, while age remained significant (*β* = −0.34, *p* = 0.017). See Table 5 for summary.

**TABLE 5:** Multivariable linear regression results—Model B.

Predictor	Regression coefficient (*β*) baseline IIEF‐6 [95% CI]	*p* value	Regression coefficient (*β*) 24‐month IIEF [95% CI]	*p* value	Regression coefficient (*β*) IIEF‐6 delta [95% CI]	*p* value
Age	**−0.34 [−0.61, −0.06]**	**0.017**	0.15 [−0.37, 0.67]	0.554	0.57 [−0.06, 1.21]	0.075
Hypertension	0.81 [−3.95, 5.56]	0.734	−2.29 [−9.14, 4.55]	0.502	−1.44 [−9.88, 7.00]	0.732
Diabetes	−4.16 [−10.7, 2.39]	0.208	−0.49 [−10.5, 9.55]	0.922	4.01 [−8.90, 16.9]	0.533
Dyslipidaemia	1.36 [−4.07, 6.79]	0.619	0.96 [−7.31, 9.24]	0.815	−3.82 [−14.6, 6.94]	0.477
Smoking	−1.84 [−6.14, 2.46]	0.395	−2.70 [−8.94, 3.54]	0.386	0.11 [−7.55, 7.77]	0.976
Body mass index	−0.51 [−1.15, 0.13]	0.118	0.09 [−1.01, 1.19]	0.866	0.77 [−0.69, 2.23]	0.291
Nerve group			5.65 [−2.57, 13.9]	0.172	5.12 [−5.57, 15.8]	0.338
APA	**5.20 [0.89, 9.51]**	**0.019**	0.03 [−6.27, 6.34]	0.991	−5.59 [−13.3, 2.16]	0.152
PER	**−0.049 [−0.089, −0.009]**	**0.017**	−0.02 [−0.077, 0.028]	0.352	0.014 [−0.051, 0.079]	0.672
CER	**0.040 [0.0001, 0.079]**	**0.049**	0.02 [−0.038, 0.081]	0.467	−0.027 [−0.100, 0.047]	0.467

*Note*: Summary of multivariable linear regression models predicting baseline erectile function (PreEF6), 2‐year erectile function (Y2EF6) and change in EF (deltEF6). Model B includes clinical predictors and imaging features (APA, PER and CER).

Abbreviations: APA, accessory pudendal artery; CER, corporal enhancement ratio; PER, prostate enhancement ratio.

For 24‐month IIEF‐6 and ΔEF6, the model incorporating imaging markers demonstrated superior AIC values compared with clinical variables only model (IIEF‐6 24 months: 383.3 vs. 422.4; ΔEF6: 395.6 vs. 446.9), although adjusted *R* ^2^ values remained low or negative. No individual variables reached statistical significance for these outcomes, although MRI‐detected APA presence demonstrated a non‐significant trend towards association with less EF decline (*β* = −5.59, 95% CI: −13.3 to 2.16, *p* = 0.152).

## 4 DISCUSSION

To our knowledge, this is the first study to evaluate a combination of preoperative mpMRI‐derived vascular markers being APA presence, prostate and corporal enhancement, for their correlation with erections after radical prostatectomy.

Our study found statistically significant difference in means of IIEF‐6 in patients with and without an MRI‐detected APA. This suggests that there is a vascular component contributing to ED after radical prostatectomy that is underrecognized. From a clinical perspective, this finding suggests that preoperative identification of APA may allow stratification of patients according to baseline vascular reserve, which could be incorporated into preoperative counselling and expectation setting. Theoretically, APA injury during surgery is thought to cause vascular compromise.^21^ Previous studies evaluating APA preservation are methodologically limited and lack integration across imaging, intraoperative assessment, postoperative vessel integrity and longitudinal functional outcomes.^13^, ^14^, ^15^ Structured MRI‐based surgical planning, including international consensus recommendations led by our group, has demonstrated that formal documentation of imaging‐informed surgical intent improves functional outcomes without compromising oncological safety.^22^ However, vascular anatomy has not yet been incorporated into this framework. The integration of vascular imaging markers into structured preoperative planning pathways may represent a logical extension of current nerve‐sparing strategies. In this context, identification of APA on routine mpMRI, as demonstrated in our study, could serve as an adjunct to guide surgical awareness of vascular anatomy, although further work is required to determine whether this is feasible and translates into improved EF recovery.

Our study identified an APA in 43.6%. This closely mirrors the existing literature, with meta‐analytic data suggesting variable detection rate of approximately 39% of men have additional arterial branches supplying the penile corpora (13). There remain uncertainties regarding the accuracy of diagnostic mpMRI in assessing pelvic vasculature and in reliably identifying APAs. To mitigate these limitations, our methodology incorporated independent review by two blinded expert uro‐radiologists.

Imaging can be used to detect APA, as demonstrated by two studies utilizing MRI angiography. When compared with intraoperative identification, MRI angiography achieved an accuracy of 92.1%; however, this showed an APA prevalence of around 20%.^23^ As the MRI has cemented its place in the diagnostics of prostate cancer, so has the quality of the images obtained, and this has been studied thoroughly and outlined in the PIQUAL classification of MRI quality.^18^ In essence, an mpMRI performed to good quality may closely represent MRI angiography as our current study suggests. There has been earlier research suggesting the importance of vascular‐sparing radical prostatectomy^9^; however, carrying out this research in the pre‐MRI era was extremely challenging, as it relied on surgeons' identification of these vessels, which has an inherent limitation. There may be now an opportunity for prospective studies evaluating whether targeted preservation of image‐identified APAs is feasible and whether this has a measurable impact on postoperative EF outcomes, potentially introducing the concept of MRI surgical planning combining nerve‐ and vascular‐sparing prostatectomy.

PER and CER were selected a priori as exploratory imaging markers because our previous systematic review identified studies reporting associations between DCE‐derived prostatic or corporal enhancement and EF.^10^, ^19^, ^20^ The present study therefore evaluated these candidate markers in an independent cohort. The proposed biological rationale for PER is indirect: Prostatic enhancement may reflect aspects of pelvic tissue contrast delivery and microvascular health that could also be associated with the vascular component of baseline EF. However, PER is not a direct measure of penile arterial inflow. Similarly, CER was derived from routine DCE imaging acquired in the flaccid state and therefore represents resting corporal contrast enhancement rather than stimulated cavernosal inflow or corporo‐venocclusive function. Although a previous study demonstrated an association between penile DCE‐derived parameters and self‐reported EF outcomes, the haemodynamic validity of CER remains unestablished. Together with the absence of associations between either enhancement ratio and 24‐month IIEF‐6 or change in IIEF‐6, these findings suggest that PER and CER have limited prognostic value and should currently be regarded as exploratory imaging measures.

Although age is an established predictor of erectile function recovery following radical prostatectomy,^24^ it was not significantly associated with 24‐month IIEF‐6 or change in IIEF‐6 in the present cohort. Interpretation of the 24‐month outcome was limited by recent sexual inactivity: Among the 56 patients with follow‐up data, 19 had not attempted intercourse during the preceding 4 weeks. As several components of the IIEF‐6 depend on recent sexual activity and intercourse attempts, scores in these patients may reflect sexual opportunity and activity in addition to physiological erectile capacity. This outcome heterogeneity, together with the smaller follow‐up sample, may have reduced the statistical power to demonstrate the expected association between age and postoperative EF. The absence of a significant association in this study should therefore be regarded as inconclusive rather than as contradicting the established prognostic importance of age.

Another fundamental limitation in interpreting the role of APA is that our study design did not ascertain whether the APA was preserved during surgery. This is due to the retrospective nature of the radiology review in our study. Therefore, while APA presence is associated with greater baseline vascular potential, its impact on postoperative recovery remains uncertain without confirmation of its intraoperative preservation or fate. However, our sample size of 71 patients is small, and larger, improved studies are needed for validation.^19^, ^20^

Although all patient data were collected prospectively, the MRI markers were reported in 2024–2025 retrospect while the RARP was performed in 2021. This retrospective analysis is a limitation in our study. Identifying differences based on vascular markers on mpMRI represents a research opportunity where more good quality prospective research is needed to validate these results and establish the significance of APA in the context of EF recovery after radical prostatectomy.

An MRI‐detected APA was independently associated with higher baseline IIEF‐6, whereas increasing age was associated with poorer baseline EF. One biologically plausible explanation is that ageing and associated vascular comorbidity may reduce pelvic and penile arterial reserve, while an APA provides an additional collateral pathway to the penile circulation and may therefore confer greater vascular redundancy, contributing to the higher baseline EF. Nevertheless, this interpretation remains hypothetical, as APA visibility on mpMRI may itself be influenced by vessel calibre, contrast opacification and overall vascular health, rather than indicating a direct protective effect of the artery. Furthermore, because intraoperative APA preservation was not documented and MRI‐detected APA was not associated with postoperative outcomes, the present study cannot establish that preserving an APA mitigates postoperative EF decline. Prospective studies integrating preoperative vascular mapping, confirmation of intraoperative APA preservation and postoperative functional vascular assessment are required to test this hypothesis.

Future research may prompt the surgeon to the MRI‐detected APA, incorporate video analysis to scrutinize APA preservation or inadvertent injury and carry out postoperative IIEF‐6 measurement to clearly outline the functional significance of the APA on EF recovery after surgery.

In summary, this study demonstrates the feasibility of using preoperative mpMRI‐derived vascular markers to assess baseline EF in patients undergoing bilateral nerve‐sparing prostatectomy. This may represent an underrecognized vascular component in the pathophysiology of post‐prostatectomy ED. While vascular indices (PER and CER) may have limited prognostic value for long‐term recovery, the identification of APA on preoperative MRI provides clinically relevant information that may support patient counselling and risk stratification in the future. These findings provide a rationale for future prospective studies evaluating MRI‐guided vascular preservation strategies and their impact on functional outcomes following radical prostatectomy.

## AUTHOR CONTRIBUTIONS

**Abdullah Al‐Mitwalli:** Conceptualization; data curation; writing—original draft; writing—review and editing. **Matthew Megson:** Data curation. **Dalia Mohammed:** Data curation. **Ahmed Al‐Nowfal:** Conceptualization; data curation; writing—review and editing. **Conrad Von Stempel:** Conceptualization; data curation; writing—review and editing. **Wai Gin Lee:** Conceptualization; supervision; writing—review and editing. **David Ralph:** Conceptualization; supervision; writing—review and editing. **Zafer Tandogdu:** Conceptualization; supervision; writing—review and editing.

## CONFLICT OF INTEREST STATEMENT

The authors do not conflict of interest to declare.

## Data Availability

The data that support the findings of this study are available from the corresponding author upon reasonable request.
